# Supplementary material for: VEGFR2 is required for VEGF-C–VEGFR3–PI3Kα-mediated sprouting lymphangiogenesis
Source: Nat Commun. 2026 May 15;17:4380. doi: 10.1038/s41467-026-73013-3 (PMC13179370; doi:10.1038/s41467-026-73013-3)
Supplement: Supplementary file 1 — Reporting Summary [file 41467_2026_73013_MOESM1_ESM.pdf]

nature portfolio

Corresponding author(s): Taija Mäkinen

Last updated by author(s): Mar 25, 2026

Reporting Summary

Nature Portfolio wishes to improve the reproducibility of the work that we publish. This form provides structure for consistency and transparency in reporting. For further information on Nature Portfolio policies, see our Editorial Policies and the Editorial Policy Checklist.

Statistics

For all statistical analyses, confirm that the following items are present in the figure legend, table legend, main text, or Methods section.

n/a	Confirmed	
☐	☒	The exact sample size (*n*) for each experimental group/condition, given as a discrete number and unit of measurement
☐	☒	A statement on whether measurements were taken from distinct samples or whether the same sample was measured repeatedly
☐	☒	The statistical test(s) used AND whether they are one- or two-sided<br>*Only common tests should be described solely by name; describe more complex techniques in the Methods section.*
☒	☐	A description of all covariates tested
☐	☒	A description of any assumptions or corrections, such as tests of normality and adjustment for multiple comparisons
☐	☒	A full description of the statistical parameters including central tendency (e.g. means) or other basic estimates (e.g. regression coefficient) AND variation (e.g. standard deviation) or associated estimates of uncertainty (e.g. confidence intervals)
☐	☒	For null hypothesis testing, the test statistic (e.g. *F*, *t*, *r*) with confidence intervals, effect sizes, degrees of freedom and *P* value noted<br>*Give P values as exact values whenever suitable.*
☒	☐	For Bayesian analysis, information on the choice of priors and Markov chain Monte Carlo settings
☒	☐	For hierarchical and complex designs, identification of the appropriate level for tests and full reporting of outcomes
☒	☐	Estimates of effect sizes (e.g. Cohen's *d*, Pearson's *r*), indicating how they were calculated

Our web collection on statistics for biologists contains articles on many of the points above.

Software and code

Policy information about availability of computer code

Data collection	Leica Application Suite (Version 4.5.0 25531 and earlier) [image aquisition]<br>BD FACSDiva Software (Version 8.0) (BD Biosciences) [flow cytometry]
Data analysis	Image J (Version 2.9.0/1.53t or earlier)<br>Adobe Photoshop (Version 27.3)<br>Graphpad Prism 9.0<br>MATLAB R2020a<br>FlowJo 10.5.0-10.5.3 (TreeStar) [flow cytometry]<br>"REAVER: A program for improved analysis of high-resolution vascular network images"<br>Corliss BA, Doty RW, Mathews C, Yates PA, Zhang T, Peirce SM.; Microcirculation.; 2020; doi:10.1111/micc.12618 [Image analysis]

For manuscripts utilizing custom algorithms or software that are central to the research but not yet described in published literature, software must be made available to editors and reviewers. We strongly encourage code deposition in a community repository (e.g. GitHub). See the Nature Portfolio guidelines for submitting code & software for further information.

Data

Policy information about availability of data

All manuscripts must include a data availability statement. This statement should provide the following information, where applicable:

- Accession codes, unique identifiers, or web links for publicly available datasets
- A description of any restrictions on data availability
- For clinical datasets or third party data, please ensure that the statement adheres to our policy

All source data supporting the quantitative findings of this study are provided as a Source Data file. All other data supporting the findings are available within the paper and its supplementary information files.

Research involving human participants, their data, or biological material

Policy information about studies with human participants or human data. See also policy information about sex, gender (identity/presentation), and sexual orientation and race, ethnicity and racism.

Reporting on sex and gender	n.a.
Reporting on race, ethnicity, or other socially relevant groupings	n.a.
Population characteristics	n.a.
Recruitment	n.a.
Ethics oversight	n.a.

Note that full information on the approval of the study protocol must also be provided in the manuscript.

Field-specific reporting

Please select the one below that is the best fit for your research. If you are not sure, read the appropriate sections before making your selection.

☒ Life sciences ☐ Behavioural & social sciences ☐ Ecological, evolutionary & environmental sciences

For a reference copy of the document with all sections, see nature.com/documents/nr-reporting-summary-flat.pdf

Life sciences study design

All studies must disclose on these points even when the disclosure is negative.

Sample size	No statistical methods were used to pre-determine sample size.<br>For all in vivo experiments, a minimum of 3 mice per condition was used, except for Fig. 4j (n=2 Vegfr2flox/flox;Vegfr3flox/flox mice). No statical testing was performed for this experiment (Fig. 4j).<br>The sample size of 3 was chosen as the minimum required to perform statistical tests.
Data exclusions	No data were excluded.
Replication	All data has been successfully replicated in at least two independent experiments.
Randomization	Allocation of mice into experimental groups was based on genotype. Littermate controls were included. Both female and male mice were included in analyses. Data were collected from different litters on different days and experiments were performed for different batches at different time points.
Blinding	No blinding was done in the data collection, analysis and quantifications. Blinding was not feasible due to visible phenotypes and genotype-dependent differences, which made group allocation apparent to the investigator. Quantification of vessel parameters presented in Figures 4 and 5 (lymphatic vessel density and branchpoints) was done in an unbiased automated fashion using ImageJ or the MATLAB script "REAVER" (Corliss, B. A. et al. Microcirc. N. Y. N 1994 27, e1261)

Reporting for specific materials, systems and methods

We require information from authors about some types of materials, experimental systems and methods used in many studies. Here, indicate whether each material, system or method listed is relevant to your study. If you are not sure if a list item applies to your research, read the appropriate section before selecting a response.

Materials & experimental systems

n/a	Involved in the study
☐	☒ Antibodies
☒	☐ Eukaryotic cell lines
☒	☐ Palaeontology and archaeology
☐	☒ Animals and other organisms
☒	☐ Clinical data
☒	☐ Dual use research of concern
☒	☐ Plants

Methods

n/a	Involved in the study
☒	☐ ChIP-seq
☐	☒ Flow cytometry
☒	☐ MRI-based neuroimaging

Antibodies

Antibodies used

The following antibodies were used for whole mount immunofluorescence (dilution 1:100-1:500): goat anti-mouse VEGFR3 (1:100) (#AF743, R&D Systems), goat anti-mouse VEGFR2 (1:100) (#AF644, R&D Systems), rat anti-mouse LYVE1 (1:300) (#AF7939, R&D systems), Rabbit anti-mouse LYVE1 (1:500) (#103-PA50AG, Reliatech), rabbit anti VEGFR2 (1:100) (#2479 Cell Signalling),rabbit anti phospho-Tyrosine (P-Tyr-1000) MultiMab (1:100) (#8954, Cell Signalling) Rat anti-mouse PECAM1 (1:200) (BD Pharmingen, 553370) Armenian hamster anti-mouse PECAM1 (1:200) (#MA3105, ThermoFisher), chicken anti-GFP (1:500) (#ab13970, Abcam), Rabbit anti-DsRed (1:500) (#632496, Takara), Syrian hamster anti-mouse PDPN (1:200) (#8.1.1, DSHB) All secondary antibodies conjugated to Cy3(JIR, 712-165-153),(JIR,711-166-152), Dylight 405 (JIR, 712-475-153), Alexa Fluor 488 (JIR, 703-545-155), (JIR, 712-545-153), (JIR, 711-545-152), Alexa Fluor 647 (JIR, 712-605-153),(JIR, 705-605-147),(JIR, 711-605-152), Cy5 (#705-175-003) or Alexa Fluor 680 (JIR, 705-625-147) were raised in donkey and obtained from Jackson ImmunoResearch (JIR); Alexa Fluor 594 (JIR, 127-585-160) was from goat. Secondary antibodies conjugated to AF405+ (# A48268), AF488+(# A48269,# A32790), AF555+ (# A32794, # A32816), AF594+ (# A32758, # A32754, # A21209) or AF647+ (# A32849, # A32795, # A32794) were obtained from ThermoFisher Scientific.

Following antibodies were used for flow cytometry:
rat anti-mouse CD16/32 antibodies (#56603, Biolegend), Syrian hamster anti-mouse PDPN-APC (#127410, Biolegend), Rat anti-mouse CD31-Pe-Cy7 (#102418, Biolegend) Rat anti-mouse LYVE1-AF488 (#53-0443-82, ThermoFisher), Rat anti-mouse CD45-eF450 (#48-0451-82, ThermoFisher), Rat anti-mouse CD11b-eF450 (#48-0112-82, ThermoFisher), Rat anti-mouse Ter119-eF450 (#48-5921-8, ThermoFisher), Rat anti-mouse CD31-PerCP-Cy5.5 (#102420, Biolegend), Syrian hamster anti-mouse PDPN-PE-Cy7 (#127412, Biolegend), Rat anti-mouse LYVE1-Biotin (#13-0443-82, Thermo Fisher), Rat anti-mouse KI67-APC (#652406, Biolegend).

Validation

Antibodies used for Immunostaining and PLA:
Armenian hamster anti-mouse PECAM1: Thermo Fisher provides several references for validation. https://www.thermofisher.com/antibody/primary/target/cd31%20(pecam-1)
chicken anti-GFP: Abcam provides several references for validation. https://www.abcam.com/gfp-antibody-ab13970.html
goat anti-mouse VEGFR3: R&D Systems provides several references for validation. https://www.rndsystems.com/products/mouse-vegfr3-flt-4-antibody_af743
goat anti-mouse VEGFR2: R&D Systems provides several references for validation.https://www.rndsystems.com/products/mouse-vegfr2-kdr-flk-1-antibody_af644
rabbit anti-mouse LYVE1: Reliatech provides several references for validation. https://www.reliatech.de/products/antibodies/polyclonal-antibodies/product/103-pa50ag/
rabbit anti-DsRed: Takara Bio provides several references for validation. https://www.takarabio.com/products/antibodies-and-elisa/fluorescent-protein-antibodies/red-fluorescent-protein-antibodies
rabbit anti-mouse VEGFR2: Cell signalling provides several references for validation. https://www.cellsignal.com/products/primary-antibodies/vegf-receptor-2-55b11-rabbit-mab/2479?srsltid=AfmBOorV_KxtOSa-5KmNrNJaWaTmXy8lIO3Y4O_Jo3gbW-EISd5qVMpK
rabbit anti phospho-Tyrosine (P-Tyr-1000) :Cell signalling provides several references for validation.https://www.cellsignal.com/products/primary-antibodies/phospho-tyrosine-p-tyr-1000-multimab-rabbit-mab-mix/8954?srsltid=AfmBOoqlQu5pdyVuZPozrFWF2AMZUcyqCMIlpVu_X-Psk_O8kwFPjZhK
rat anti-mouse PECAM1: BectonDickinson provides several references for validation. https://www.bdbiosciences.com/eu/applications/research/stem-cell-research/cancer-research/mouse/purified-rat-anti-mouse-cd31-mec-133/p/553370
rat anti-mouse LYVE1: R&D Systems provides several references for validation. https://www.rndsystems.com/products/mouse-lyve-1-antibody-223322_mab2125
syrian hamster anti-mouse PDPN: DSHB provides several references for validation. https://dshb.biology.uiowa.edu/8-1-1

Antibodies used for flow cytometry:
rat anti-mouse KI67-APC: Biolegend provides several references for validation. https://www.biolegend.com/nl-be/products/apc-anti-mouse-ki-67-antibody-8447
rat anti-mouse LYVE1-Biotin: Thermo Fisher provides several references for validation. https://www.thermofisher.com/antibody/product/LYVE1-Antibody-clone-ALY7-Monoclonal/13-0443-82
rat anti-mouse CD16/CD32: eBioscience provides several references for validation. https://www.thermofisher.com/antibody/product/CD16-CD32-Antibody-clone-93-Monoclonal/14-0161-82
syrian hamster anti-mouse PDPN-APC: Biolegend provides several references for validation
https://www.biolegend.com/nl-be/products/apc-anti-mouse-podoplanin-antibody-6656?GroupID=BLG5772
rat anti-mouse CD31-Pe-Cy7: Biolegend provides several references for validation. https://www.biolegend.com/fr-ch/products/pe-cyanine7-anti-mouse-cd31-antibody-3942
rat anti-mouse LYVE1-AF488: Thermo Fisher provides several references for validation. https://www.thermofisher.com/antibody/product/LYVE1-Antibody-clone-ALY7-Monoclonal/53-0443-82
rat anti-mouse CD45-eF450: Thermo Fisher provides several references for validation. https://www.thermofisher.com/order/genome-database/generatePdf?productName=CD45&assayType=ANTIBODY&productId=48-0451-80&detailed=true
rat anti-mouse CD11b-eF450: Thermo Fisher provides several references for validation. https://www.thermofisher.com/antibody/

product/CD11b-Antibody-clone-M1-70-Monoclonal/48-0112-82
rat anti-mouse Ter119-eF450: Thermo Fisher provides several references for validation. https://www.thermofisher.com/antibody/product/TER-119-Antibody-clone-TER-119-Monoclonal/48-5921-82
rat anti-mouse CD31-PerCP-Cy5.5 : Biolegend provides several references for validation. https://www.biolegend.com/en-gb/products/percp-cyanine5-5-anti-mouse-cd31-antibody-6668?GroupID=BLG1566
syrian hamster anti-mouse PDPN-PE-Cy7: Biolegend provides several references for validation. https://www.biolegend.com/fr-lu/products/pe-cyanine7-anti-mouse-podoplanin-antibody-6674

Animals and other research organisms

Policy information about studies involving animals; ARRIVE guidelines recommended for reporting animal research, and Sex and Gender in Research

Laboratory animals	PGK-Cre (Lallemand et al, 1998), Prox1-CreERT2 (Bazigou et al, 2011), Vegfr1-CreERT2 (Petkova et al, 2023), Vegfr3-CreERT2 (Martinez-Corral et al, 2016), R26-iMb2-Mosaic (Pontes-Quero et al, 2017), R26-iMb-VEGFR2 (Pontes-Quero et al, 2017), Kdr-flox(tm1Wag) (Haigh et al.,2003), Kdr-flox(tm2Sato) (Hooper et al, 2009), Flt4-flox (Haiko et al, 2008), R26-LSL-Pik3ca-H1047R (Eser et al, 203), Pik3ca-flox (Graupera et al, 2008), R26-mTmG (Muzumdar et al, 2007), iSuRe-Cre (Fernandez-Chacon et al, 2019), and R26-iSuRe-HadCre (Garcia-Gonzalez et al, 2024) were all analyzed on a C57BL/6J background. Both female and male mice were used for analysis and no differences in the phenotype between the sexes were observed. The age is stated in the figures and/or legends. Mice were housed in individually ventilated cages (GM500, Tecniplast) under a 12:12-h dark–light cycle (light from 07:00 to 19:00) at 22 ± 1°C under 40-60% humidity with ad libitum access to food and water.
Wild animals	The study did not involve wild animals.
Reporting on sex	Both female and male mice were included in analyses. No differences in the phenotype between the sexes were observed. Sex of mice is provided in source data were applicable.
Field-collected samples	The study did not involve samples collected from the field.
Ethics oversight	All experimental procedures were approved by the Uppsala Laboratory Animal Ethical Committee, Sweden, or the Finnish Project Authorisation Board (Eläinkoelautakunta ELLA)

Note that full information on the approval of the study protocol must also be provided in the manuscript.

Plants

Seed stocks	n/a
Novel plant genotypes	n/a
Authentication	n/a

Flow Cytometry

Plots

Confirm that:

☒ The axis labels state the marker and fluorochrome used (e.g. CD4-FITC).

☒ The axis scales are clearly visible. Include numbers along axes only for bottom left plot of group (a 'group' is an analysis of identical markers).

☒ All plots are contour plots with outliers or pseudocolor plots.

☒ A numerical value for number of cells or percentage (with statistics) is provided.

Methodology

Sample preparation	For FACS analysis of LYVE1 populations ar skins were dissected, cut into small pieces and digested in Collagenase IV (Life Technologies) 10 mg/ml, DNase1 (Roche) 0.1 mg/ml and FBS 0.5 % (Life Technologies) in PBS at 37 °C for 30 min. Collagenase

activity was quenched by dilution with FACS buffer (PBS, 0.5 % FBS, 2 mM EDTA) and digestion products were filtered twice through 70 µm nylon filters (BD Biosciences). Cells were washed with FACS buffer and immediately processed for immunostaining first by blocking Fc receptor binding with rat anti-mouse CD16/CD32 followed by incubation with antibodies targeting PDPN, LYVE1, CD31/PECAM1, CD45 and CD11b.
For FACS analysis of proliferating cells ear skins were dissected, cut into small pieces and digested in Collagenase IV (Life Technologies) 10 mg/ml, DNase1 (Roche) 0.1 mg/ml and FBS 0.5 % (Life Technologies) in PBS at 37 °C for 30 min. Collagenase activity was quenched by dilution with FACS buffer (PBS, 0.5 % FBS, 2 mM EDTA) and digestion products were filtered twice through 70 µm nylon filters (BD Biosciences). Cells were washed with FACS buffer and immediately processed for immunostaining first by blocking Fc receptor binding with rat anti-mouse CD16/CD32 followed by incubation with antibodies targeting PDPN, CD31/PECAM1, CD45 and CD11b. After staining, cells were washed with PBS and then stained for dead cells using the blue LIVE/DEAD® fixable dead cell stain kit (Life Technologies), followed by fixation and permeabilization using the Foxp3/Transcription factor staining kit according to the manufacturer's instructions. Finally cells were incubated with rat serum and KI67 antibody.

Instrument

Cells were analyzed on a BD LSR Fortessa cell analyzer equipped with 5 lasers (355, 405, 488, 561 and 643 nm)

Software

FlowJo software version 10.5.0-10.5.3 (TreeStar)

Cell population abundance

Analysis of LYVE1 population: ECs (PECAM1+) of total cells 1.5-2%; LECs (PDPN+) of ECs 20-30%, LYVE1-low: 18.9-31.3%, LYVE1-intermediate: 15.3-22.3%, LYVE-high: 50.6%-60.9%
Analysis of proliferating LECs: ECs (PECAM1+) of total cells 1.9-5%; LECs (PDPN+) of ECs 25.4-74.4%, KI67+ LECs of all LECs 4.8-30.5%.

Gating strategy

Single viable cells were gated from FSC-A/SSC-A, FSC-H/FSC-W and SSC-H/SSC-W plots followed by exclusion of dead cells in the UV dump channel. FMO controls were used to set up the subsequent gating scheme to obtain cell populations and quantification of proliferating cells. Gating was done as described and exemplified in Martinez-Corral et al, Nat Commun 2020.

☐ Tick this box to confirm that a figure exemplifying the gating strategy is provided in the Supplementary Information.
