# Supplementary material for: VEGFR2 is required for VEGF-C–VEGFR3–PI3Kα-mediated sprouting lymphangiogenesis
Source: Nat Commun. 2026 May 15;17:4380. doi: 10.1038/s41467-026-73013-3 (PMC13179370; doi:10.1038/s41467-026-73013-3)
Supplement: Supplementary file 3 — Supplementary Information [file 41467_2026_73013_MOESM3_ESM.pdf]

Supplementary Information

VEGFR2 is required for VEGF-C–VEGFR3–PI3Kα-mediated sprouting lymphangiogenesis

Schoofs H, Zhang Y, Ortsäter H, Lytvyn M, Benedito R, and Mäkinen T*

*Corresponding author: E-mail: taija.makinen@helsinki.fi

Inventory of Supporting Information

Supplementary Fig. 1. Efficient deletion of *Vegfr2* in BECs without alterations in blood vessel morphology.

Supplementary Fig. 2. Faithful tracking of *Vegfr2*-deleted LECs in the postnatal dermal vasculature.

Supplementary Fig. 3. Efficient deletion of *Vegfr2* and *Vegfr3* using the *R26-iSuRe-HadCre* allele.

Supplementary Fig. 4. Early effects of VEGF-C stimulation and PI3Kα activation on lymphatic capillaries.

Supplementary Fig. 5. Assessment of the roles of VEGFR2 and VEGFR3 in regenerative lymphangiogenesis.

Supplementary Fig. 6. Validation of unpermeabilized tissue staining and a whole-mount PLA protocol.

Supplementary Fig. 7. Lack of effect of LEC-specific *Prox1-CreER*^{T2}-mediated deletion of *Pik3ca* on VEGFR2 levels on blood vessel endothelium.

Supplementary Figures

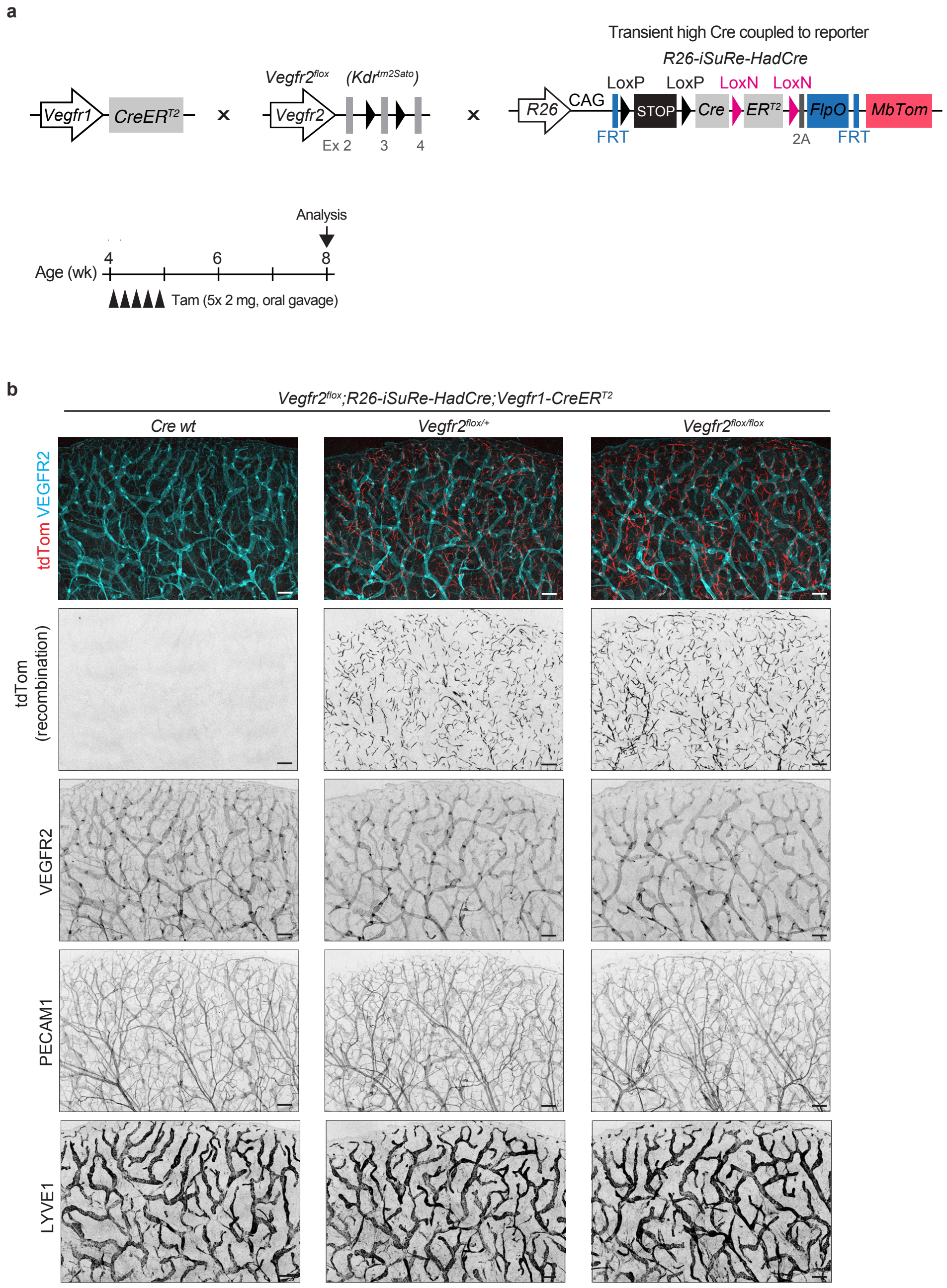

Supplementary Fig. 1. Efficient deletion of *Vegfr2* in BECs without alterations in blood vessel morphology. (**a**) Experimental scheme and genetic constructs for efficient deletion of *Vegfr2* in BECs using the *Vegfr1-CreER^{T2}* transgene. (**b**) Whole mount immunofluorescence of mouse ear skin in 8-week-old mice, conducted two weeks after inducing *Vegfr2* deletion, showing efficient depletion of VEGFR2 in BECs, even in the absence of recombination of the *R26-iSuRe-HadCre* allele, but not in LECs. Number of mice analyzed per genotype: n=6 (Cre wt ctrl), n=4 (flox/+), n=5 (flox/flox). Scale bars: 200 µm (b).

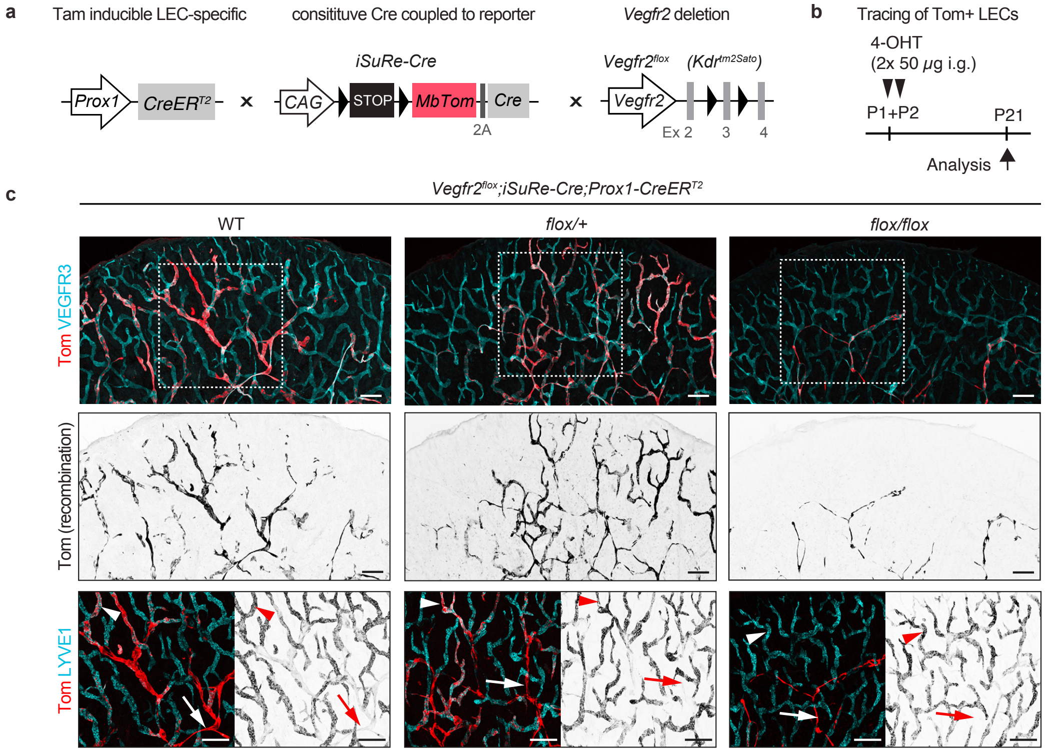

Supplementary Fig. 2. Faithful tracking of *Vegfr2*-deleted LECs in the postnatal dermal vasculature. (**a**,**b**) Genetic constructs (**a**) and experimental scheme (**b**) for faithful tracking of *Vegfr2*-deleted LECs using the *iSuRe-Cre* allele that switches upon conditional $CreER^{T2}$ activation to the expression of constitutive Cre and MbTomato reporter. (**c**) Whole mount immunofluorescence showing the distribution of Tom^{+} LECs, labelled at early postnatal development, within the mature dermal vasculature in 3-week-old mice. Cre^{+} mice carrying solely *iSuRe-Cre* or *iSuRe-Cre* and heterozygous (*flox/+*) *Vegfr2* allele show contribution of Tom^{+} LECs in both lymphatic capillaries (arrowhead) and collecting vessels (arrow), while *Vegfr2*-deleted (*flox/flox*) mice show labelling predominantly in collecting vessels (arrow). Boxed areas are magnified to show the absence of Tom^{+} cells in lymphatic capillaries of $Vegfr2^{flox/flox}$ mice. Scale bars: 250 µm (c).

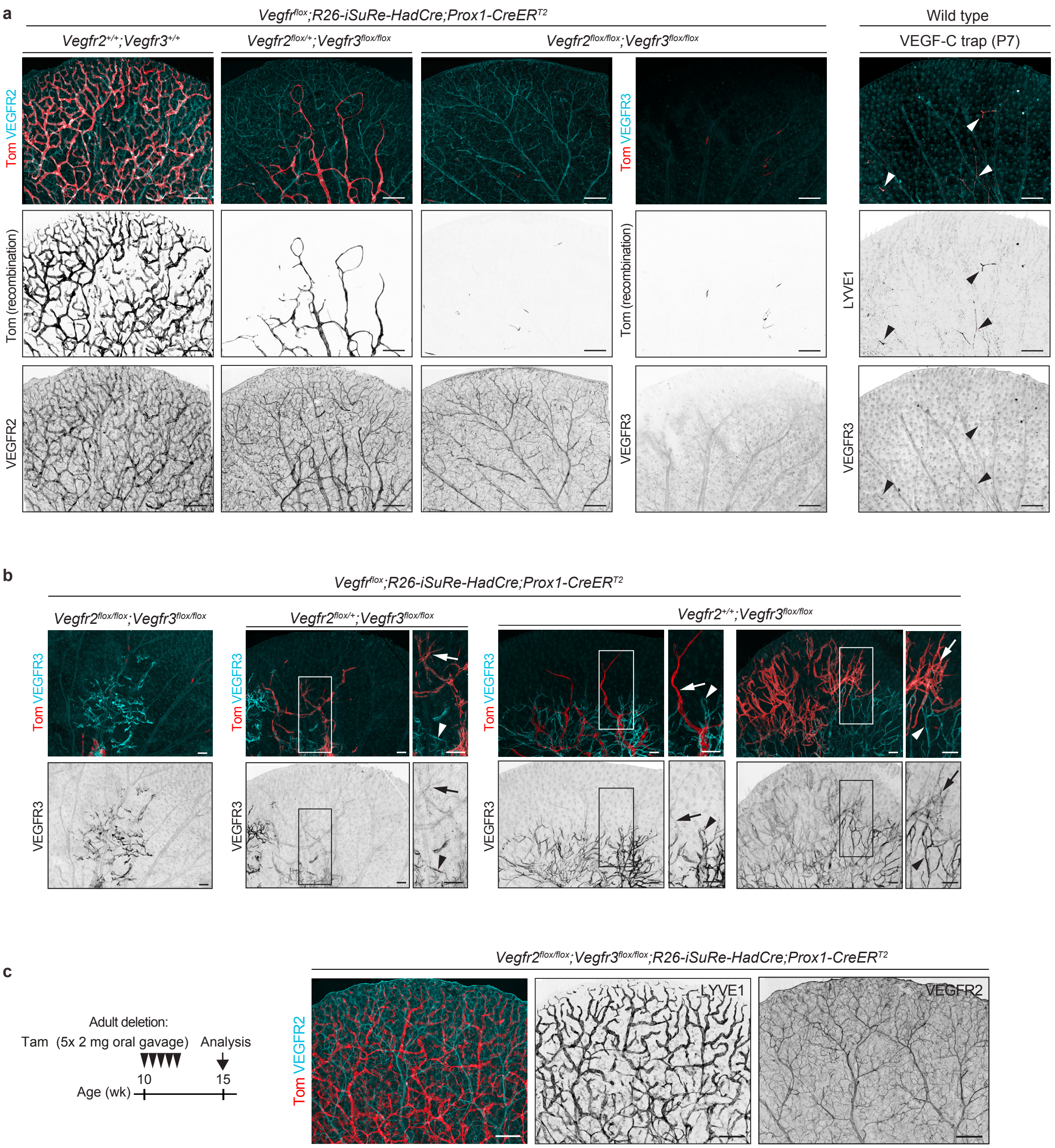

Supplementary Fig. 3. Efficient deletion of *Vegfr2* and *Vegfr3* using the *R26-iSuRe-HadCre* allele. (**a**) Additional single-channel images from **Fig. 4j** showing efficient depletion of VEGFR2 and VEGFR3 and loss of lymphatic vessels in *Vegfr2*$^{flox/flox}$;*Vegfr3*$^{flox/flox}$;*R26-iSuRe-HadCre;Prox1-CreER*^{T2} mice. Postnatal VEGF-C inhibition by administration of AAV9 encoding soluble VEGF-C trap at postnatal day 7 (P7) also shows loss of most lymphatic vessels by P21, with only small vessel remnants remaining (arrowheads). (**b**) Examples of LECs escaping *R26-iSuRe-HadCre;Prox1-CreER*^{T2}-mediated recombination and retaining *Vegfr2* and/or *Vegfr3* expression, leading to abnormally patterned lymphatic vessels in mice of the indicated genotypes. Recombined Tom^{+} vessels retaining low VEGFR3 expression (arrows), compared to unrecombined vessels (arrowheads), suggesting deletion of only one *Vegfr3*^{flox} allele. Boxed areas are magnified. (**c**) Adult deletion of VEGFRs in 10-week-old *Vegfr2*$^{flox/flox}$; *Vegfr3*$^{flox/flox}$;*R26-iSuRe-HadCre;Prox1-CreER*^{T2} mice does not lead to vascular defects within 5-week analysis period. Scale bar: 250 µm (a-c).

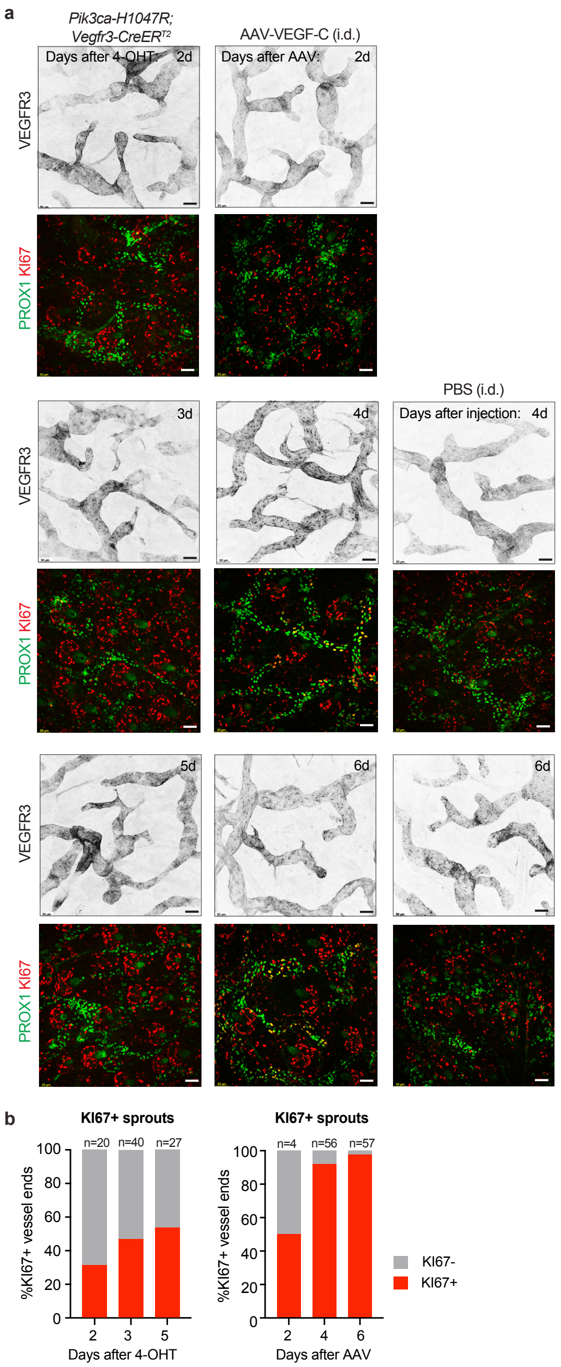

Supplementary Fig. 4. Early effects of VEGF-C stimulation and PI3Kα activation on lymphatic capillaries. (a) Additional early time point, lower magnification images corresponding to analysis shown in **Fig. 5h,I**, showing lymphatic capillary morphology and LEC proliferation (KI67+PROX1+) in AAV-VEGF-C-treated mice and in *Pik3ca*^{H1047R};*Vegfr3-CreER*^{T2} mice following topical application of 4-OHT to activate PI3Kα. PBS injection is shown as a control for AAV administration, and resulted in transient increase in vessel sprouts and LEC proliferation, localized to the injection site, but no overall effects at the stages relevant for phenotype analysis (>4 days). (**b**) Quantification of LEC proliferation in lymphatic sprouts shows induction of sprouting accompanied by robust LEC proliferation in both conditions. 3-5 mice were analyzed for each time point, and the number of vessel ends analyzed is indicated. Scale bar: 50 µm (a).

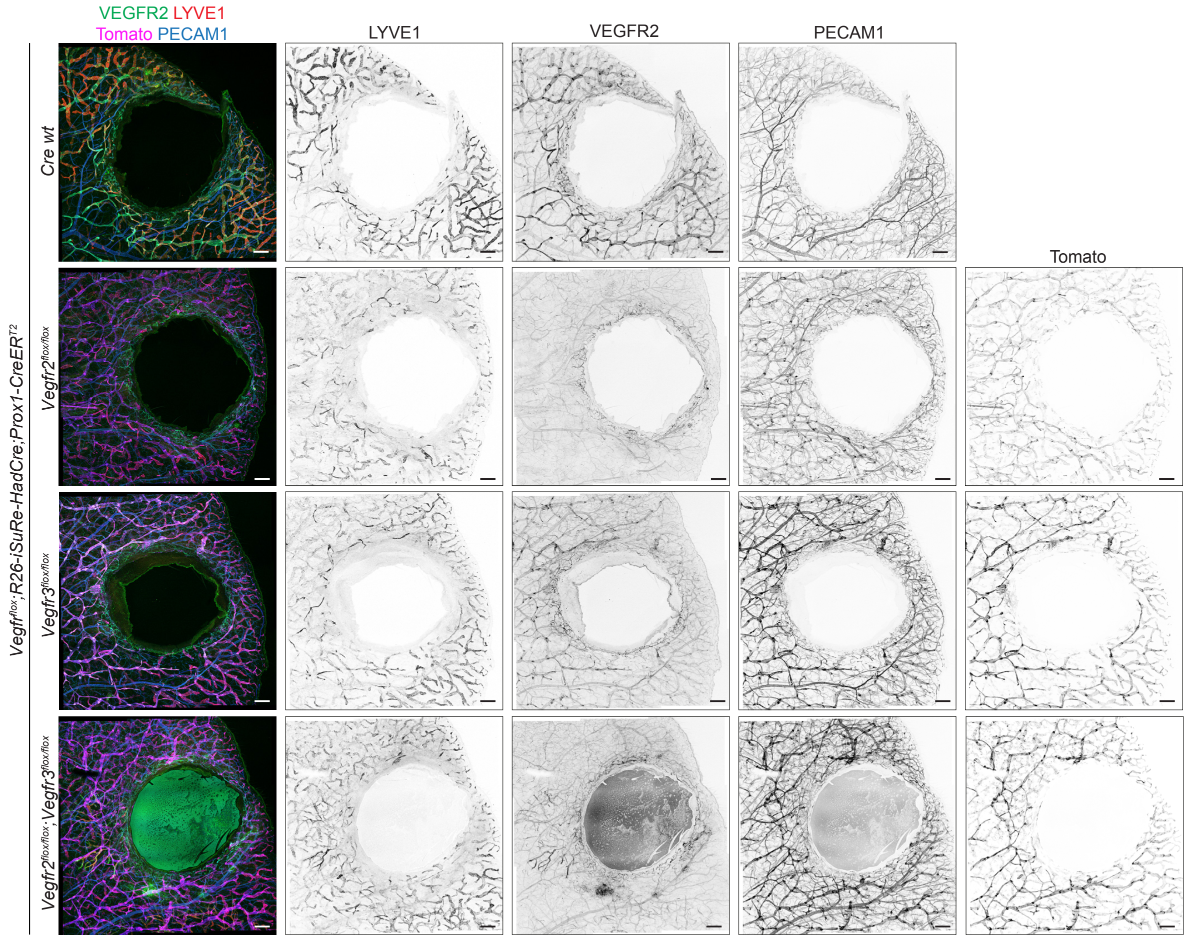

Supplementary Fig. 5. Assessment of the roles of VEGFR2 and VEGFR3 in regenerative lymphangiogenesis. Whole mount immunofluorescence of punched ear skin in *Vegfr^{flox};R26-iSuRe-HadCre;Prox1-CreER^{T2}* mice, showing vessel regrowth at the wound edge one week after injury. Lower-magnification images of tissues stained with the indicated antibodies, corresponding to those shown in **Fig. 6**, are shown. Scale bar: 250 µm.

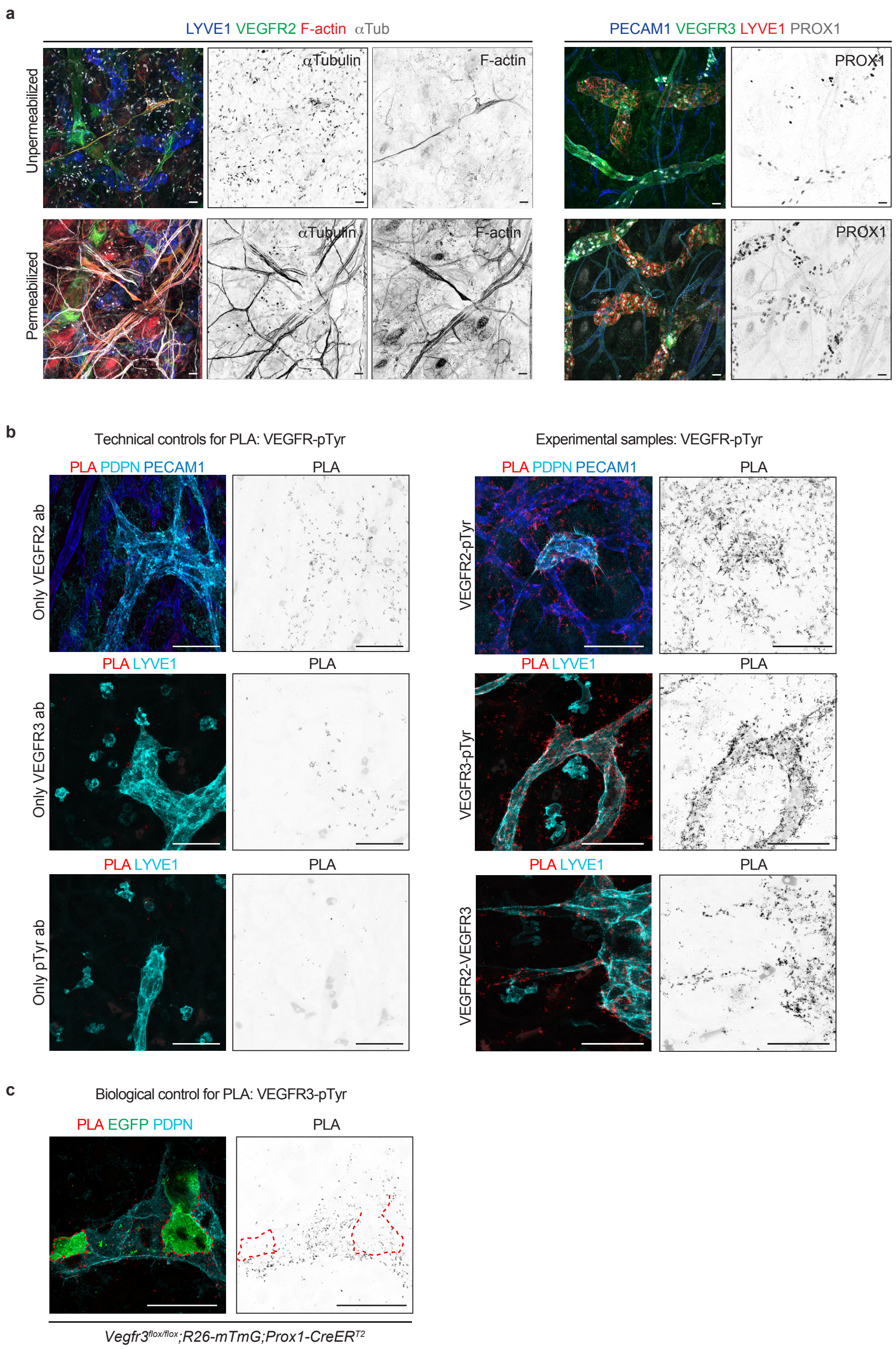

Supplementary Fig. 6. Validation of unpermeabilized tissue staining and a whole-mount PLA protocol. (**a**) Whole-mount immunofluorescence staining of unpermeabilized and permeabilized adult ear skin tissue using the indicated antibodies. Limited staining of intracellular proteins α-tubulin and F-actin (left panels), as well as PROX1 (right panels), is observed in unpermeabilized tissue. Note the unspecific staining from secondary (anti-mouse IgG) antibodies in α-tubulin staining, and weak F-actin/PROX1 signals detected in a small subset of cells in unpermeabilized tissue, likely due to partial membrane disruption. (**b**) Comparison of technical controls for whole mount PLA assay in E15 skin versus experimental samples. Only one antibody was used for PLA signal development in technical control (left) whereas both antibodies were present in experimental samples (right),

followed by co-staining with antibodies against PDPN and PECAM1, or LYVE1. Note neglectable PLA signal in technical controls as compared to experimental samples. (**c**) Biological control for the specificity of VEGFR3-pTyr PLA, assessed in E15 skin after mosaic LEC-specific genetic deletion of *Vegfr3*, reported by GFP expression (driven by the *R26-mTmG* allele) and co-stained with antibodies against PDPN. Note negligible PLA signal in recombined cells (red dotted line) but not in neighboring wild type cells. Scale bar: 25 µm (a), 50 µm (b,c).

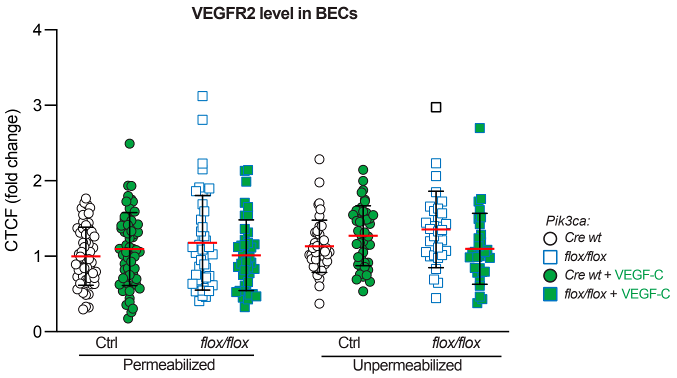

Supplementary Fig. 7. Lack of effect of LEC-specific *Prox1-CreER*^{T2}-mediated deletion of *Pik3ca* on VEGFR2 levels on blood vessel endothelium. Cre-mediated recombination was induced at 3 weeks of age by five consecutive daily administrations of tamoxifen (1 mg). Two weeks later, mice received an intradermal injection of AAV9-VEGF-C into the ear skin and were analyzed after an additional two weeks. Quantification of VEGFR2 levels within blood vessels is represented as corrected total cell fluorescence (CTCF) normalized to control values (n=120 measurements [4 mice] (Cre wt ± VEGF-C, permeabilized); n=90 measurements [3 mice] (flox/flox – VEGF-C, permeabilized); n=80 measurements [3 mice] (flox/flox + VEGF-C, permeabilized); n=90 measurements [3 mice] (Cre wt ± VEGF-C, unpermeabilized); n=60 measurements [2 mice] (flox/flox ± VEGF-C, unpermeabilized) and shown as mean ± s.d. All not significant; One-way ANOVA followed by Tukey's multiple comparison test.
